# Los delirios de Felipe Raigosa

**DOI:** 10.1590/S0104-59702025000100011

**Published:** 2025-04-07

**Authors:** Andrés Ríos Molina

**Affiliations:** i Instituto de Investigaciones Históricas, Universidad Nacional Autónoma de México. Ciudad de México – México, orcid.org/0000-0002-6133-478X, andresriosmolina@gmail.com

**Figure:**
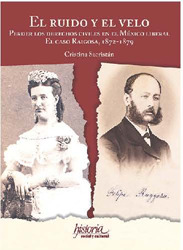
SACRISTÁN, Cristina. *El ruido y el velo: perder los derechos civiles en el México liberal. El caso Raigosa, 1872-1879*. Ciudad de México: Instituto de Investigaciones José María Luis Mora; Fondo de Cultura Económica, 2024. 357p.

Felipe Raigosa conoció la riqueza y el poder, pero terminó sus días en el ostracismo y la locura. Fue un abogado que nació en la pobreza en un pequeño pueblo mexicano, pero, gracias a su tesón y arduo trabajo, logró colarse entre la élite con una profesión de reconocido prestigio. Se casó con Manuela Moncada, quien era descendiente de uno de los hombres más ricos de México: el marqués de Jaral del Berrio. Ella era 20 años menor que él y, como solía ser en la época, el matrimonio fue arreglado: resultaba ideal la alianza entre una adinerada descendiente de la aristocracia local, con un joven abogado de notable reputación entre la élite política. Los mejores días llegaron con el Segundo Imperio. El gobierno de Maximiliano de Habsburgo (1863-1867) posicionó en la corte a una élite conservadora, que se afanó por conseguir documentación para demostrar su noble origen. Manuela llegó a ser una de las damas de compañía de la emperatriz Carlota, y Felipe fue nombrado ministro de Justicia. Desde allí disfrutaron los privilegios económicos, políticos y sociales de formar parte de la élite imperial. Pero los días de jauja duraron poco. El Imperio cayó en manos de la República liberal liderada por Benito Juárez, y quienes apoyaron el gobierno extranjero llegaron a los tribunales. Raigosa pasó tres años en la cárcel y ahí iniciaron sus penurias. Cuando recobró la libertad, Manuela decidió irse de la casa con los hijos, se llevó todos los objetos de valor e inició un juicio de interdicción para quitarle a Felipe el control sobre las propriedades, ya que, según ella, él presentaba síntomas de enajenación mental. Participaron como peritos 16 reconocidos médicos, aunque solo dos de ellos tenían experiencia en el tratamiento de la locura: Miguel Alvarado y José Peón Contreras. El libro de Cristina Sacristán (2024) reconstruye con lujo de detalles la historia de Felipe Raigosa y el complejo proceso jurídico en que se vio envuelto, desde la primera detención solicitada por el Ministerio Público, hasta su muerte en el Hospital San Hipólito para hombres dementes. La autora recopila numerosas fuentes que van desde los peritajes psiquiátricos, documentación relacionada con el proceso jurídico, artículos periodísticos donde aparece el seguimiento al “escandaloso” caso, y una gran cantidad de documentos de archivo que reconstruyen detalladamente los avatares jurídicos y psiquiátricos del Raigosa.

*El ruido y el velo* es un libro escrito con una fluidez narrativa poco común en el mundo académico: más de 300 páginas que atrapan la atención del lector gracias a la elegante y magistral prosa de Cristina Sacristán. Como bien se advierte en la introducción, la autora sigue los pasos de Carlo Ginzburg en *El queso y los gusanos*, lo que le permite, por una parte, darles a los detalles, aparentemente marginales, un lugar en un marco de significados locales y, por otra, interpretar el caso en un amplio proceso estructural: las implicaciones que tuvo en la vida familiar el cambio de régimen jurídico propiciado con la codificación del derecho. Dos años antes de que comenzara el proceso contra Raigosa, se emitió el Código Civil (1870), el cual tipificaba el juicio de interdicción como la herramienta jurídica para que las familias defendieran su patrimonio en caso de que el encargado de su manejo enloqueciera y dilapidara los bienes. El interdicto, una vez demostrada su locura, perdía todos sus derechos civiles: no podía comerciar, firmar contratos, comprar o vender propiedades, tener cargos públicos, ni siquiera el cuidado de sus hijos, y se le nombraba un tutor que lo representara. Esto implicaba imponer a los supuestos locos el mismo tutelaje asignado a los niños. En casos donde la discapacidad intelectual era marcada, no era problemático que un tutor se encargara de tomar decisiones. Pero en el caso de Raigosa, donde la “locura” siempre se puso en duda, la interdicción obró en beneficio de la familia Moncada y en perjuicio absoluto de Felipe, quien quedó en un estado de total vulnerabilidad y, además, despojado de sus derechos civiles. Después de haber sido ministro de Justicia, el juicio de interdicción le quitó la posibilidad de ejercer como abogado, administrar las propiedades, además de cuidar y mantener a sus hijos. Sacristán nos muestra que la figura creada para defender el patrimonio familiar tenía un lado oscuro: quitarle al loco todos sus derechos civiles y lanzarlo a la indefensión jurídica.

El caso estuvo lleno de irregularidades. Para comenzar, los peritajes estuvieron muy lejos del profesionalismo que se esperaba: los primeros cuatro ni siquiera hicieron una valoración clínica a Raigosa; más bien, fueron hechos a partir de las descripciones que la familia Moncada hizo de las “locuras” de Felipe. Además, los diagnósticos hechos (manía y megalomanía) evidenciaban un desconocimiento de criterios clínicos más refinados para el análisis de los delirios y utilizados en otras partes de la región, como los propuestos por Jules Falret y Valentin Magnan (Sozzo, 2015). Los síntomas de locura tampoco eran del todo claros: gastaba mucho dinero en libros de medicina, lo cual no era “normal”, según los familiares de Manuela; cuando salió de prisión hizo regalos excesivamente generosos a los vecinos de su hacienda; hizo un inventario de todos los bienes de dicha hacienda, pero de manera tan detallada que fue considerada como exagerada; tenía escrita una receta contra malos partos que había conseguido de un curandero, la cual fue interpretada como una manifestación de locura por los médicos peritos; debido a un altercado con unos vecinos, optó por tener una espada para defenderlos, razón por la que se afirmó que era “violento”, además, compró costales para esconder a los hijos en caso de que entraran a la casa a atacarlos, y la lista de excentricidades continúa. En los periódicos se decía que obligaba a los hijos a comer carne cruda y “los hacía beber hasta embriagarse”. Todo se explicaba por haber sufrido erisipela en su juventud, afección que produjo eventuales accesos febriles. Raigosa, como buen abogado y haciendo gala de una gran lucidez, cuestionó los peritajes y el proceso. ¿Por qué el interés de Manuela por demostrar la locura de su esposo y tramitar la interdicción? Todo apuntaba a que Felipe pensaba demandar a la familia Moncada porque no le habían dado a su esposa la herencia que le correspondía, de manera que es muy probable que haya sido manipulada por la familia para impedir cualquier querella por ese patrimonio. Por consiguiente, lejos de posicionar a la psiquiatría como saber científico sobre las enfermedades de la mente ante la opinión pública, este caso evidenció que la ciencia y sus representantes podían replegarse ante los poderosos intereses familiares.

Después de un largo y atropellado proceso lleno de irregularidades, la mente de Raigosa estalló. Elaboró un conjunto de textos delirantes que fueron presentados como pruebas irrefutables de locura; etapa que coincidió con la muerte de su hijo mayor a los 12 años de edad. En un texto reconstruyó su propia genealogía, donde se presentaba como descendiente de nobles familias de Persia y protagonista de una vida definida por la voluntad divina. Él, un hombre sin abolengo alguno, deliraba con la grandeza que le otorgaba una real prosapia. ¿Cómo abordar el delirio en un caso como este? Sin ánimo de imprimir un diagnóstico en retrospectiva, Cristina Sacristán hace una interesante reflexión a partir de la mirada psicoanalítica que aborda el delirio como un mecanismo a través del cual el sujeto comienza a reestructurar el sentido de su vida (Colina, 2001). El abordaje de la subjetividad de Raigosa fue posible gracias a que hubo una extensa y detallada investigación histórica que permitió comprender a fondo el sujeto, su trayectoria y los problemas que tuvo que enfrentar. En un contexto historiográfico que ha priorizado el abordaje de la subjetividad de los pacientes mentales, este libro es una lección metodológica para el análisis de textos delirantes a partir de un conocimiento riguroso de la historia de vida del sujeto.

*El ruido y el velo* es un referente obligado para quienes estén interesados en la historia de la psiquiatría, del derecho, de la subjetividad y de la familia. Esta obra, que se coció a fuego lento a lo largo de varios años de investigación y reflexión, ahora llega a manos del público lector como una enseñanza de rigor analítico y elegante escritura.
